# Rationalising drug delivery using nanoparticles: a combined simulation and immunology study of GnRH adsorbed to silica nanoparticles

**DOI:** 10.1038/s41598-018-35143-7

**Published:** 2018-11-20

**Authors:** David J. Connell, Ayman Gebril, Mohammad A. H. Khan, Siddharth V. Patwardhan, Karina Kubiak-Ossowska, Valerie A. Ferro, Paul A. Mulheran

**Affiliations:** 1 Department of Chemical and Process Engineering, University of Strathclyde, 75 Montrose Street, Glasgow, G1 1XJ UK; 2 Strathclyde Institute of Pharmacy and Biomedical Sciences, University of Strathclyde, 161 Cathedral Street, Glasgow, G4 0RE UK; 3 Department of Pathology, Faculty of Veterinary Science, Bangladesh Agricultural University, Mymensingh, 2202 Bangladesh; 4 Department of Chemical and Biological Engineering, The University of Sheffield, Western Bank, Sheffield, S10 2TN UK

## Abstract

Silica nanoparticles (SiNPs) have been shown to have significant potential for drug delivery and as adjuvants for vaccines. We have simulated the adsorption of GnRH-I (gonadotrophin releasing hormone I) and a cysteine-tagged modification (cys-GnRH-I) to model silica surfaces, as well as its conjugation to the widely-used carrier protein bovine serum albumin (BSA). Our subsequent immunological studies revealed no significant antibody production was caused by the peptide-SiNP systems, indicating that the treatment was not effective. However, the testosterone response with the native peptide-SiNPs indicated a drug effect not found with cys-GnRH-I-SiNPs; this behaviour is explained by the specific orientation of the peptides at the silica surface found in the simulations. With the BSA systems, we found significant testosterone reduction, particularly for the BSA-native conjugates, and an antibody response that was notably higher with the SiNPs acting as an adjuvant; this behaviour again correlates well with the epitope presentation predicted by the simulations. The range of immunological and hormone response can therefore be interpreted and understood by the simulation results and the presentation of the peptides to solution, paving the way for the future rational design of drug delivery and vaccine systems guided by biomolecular simulation.

## Introduction

The use of nanoparticles, and silica nanoparticles (SiNPs) in particular, as effective drug delivery vehicles has been the topic of much recent research^1–4^. Silica has excellent biodegradable properties and is generally recognised as safe for use in cosmetics and food additives, and SiNPs offer control of drug loading and release^5^. SiNPs have also been used for antigen carriers and adjuvants for vaccine delivery^3^. Since there is a reported decline in the development of new effective vaccines against a number of major diseases as well as novel targets such as cancer^6^, it is imperative that we obtain a better understanding of how the drug delivery systems work and therefore how to design new and effective vaccine systems with improved antigen presentation^7^.

In this study we used SiNPs loaded with the model peptide gonadotrophin releasing hormone I (GnRH-I) and a cysteine-tagged modified peptide (cys-GnRH-I), an established antigen developed for contraceptive use^8^. In order to gain new understanding into how this model system behaves at the molecular level, we performed fully atomistic biomolecular simulations of the peptide interactions with a model silica surface. We then performed immunisation studies, and aim to interpret the results of these using the structural details provided by the simulation. In this way, we hope to demonstrate not only the power of simulation to provide new understanding for the interpretation of experimental results, but also its potential to guide the future formulation of effective drug delivery and adjuvant systems. The modelling work is not restricted to SiNPs, and indeed it shows that other materials with similar physico-chemical properties could be used. The advantages of such an *in silico* design strategy include economic savings of labour and materials; the ethical reduction in animal studies; and most crucially a molecular-scale insight into key processes that is not achievable in any other way.

GnRH-I is a decapeptide that is produced by the hypothalamus and stimulates the release of follicle-stimulating hormone (FSH) and luteinising hormone (LH) in females and males. As such, GnRH-I effectively regulates fertility^9^, and immunisation against GnRH-I can have a major effect on the fertility of both sexes in mammals^10–15^. This is caused by antibodies, induced by the vaccine, neutralising any circulating GnRH-I, thus preventing it from stimulating FSH and LH secretion^8^. However, since GnRH-I is a naturally occurring self-peptide, it needs to be conjugated to a foreign carrier protein to induce the required immune response^13^. Indeed, with peptide vaccines an adjuvant is very often crucial to stimulate the immune response to adequate levels.

We used 200 nm Stöber SiNPs as the adjuvant, since these have proven their efficacy in other vaccine studies^1,2,16–19^. The interaction of proteins and peptides with inorganic materials has been studied using fully atomistic molecular dynamics (MD) simulations by a number of groups^20–28^. For example, model silica surfaces have been used to understand and interpret experimental lysozyme adsorption^23^, as well as the functionalisation of SiNPs with cell penetrating peptides^22^. In this work we used our simulation approach^29^ to investigate how the GnRH-I and cys-GnRH-I peptides interact with the model silica surface. We also simulated the behaviour of cys-GnRH-I covalently bonded to the surface of the carrier protein bovine serum albumin (BSA). With these simulations, we obtained novel insight into the morphological arrangements of the peptides at the adjuvant surfaces, and how key epitopes are presented to the biological environment.

In our experiments, we explored the effect of Stöber SiNPs and BSA, both separately and combined, as immunopotentiators in combination with GnRH-I and cys-GnRH-I. The BSA and SiNPs both acted as foreign molecules to trigger the immune system into a response. The effectiveness of the response was assessed and compared by measuring the GnRH-specific antibody and testosterone levels in male mice that received the different vaccination formulations subcutaneously over a period of 13 weeks. We also investigated the impact of the treatments on spermatogenesis. We found that the various treatments induced different responses, and importantly, we were able to interpret and understand the results with reference to the simulation studies. We believe that this paves the way for new possibilities using simulation tools to design new vaccine formulations and thereby greatly reduce the cost and time of the experimental phase of vaccine development.

## Results

### Molecular Dynamics Simulations (MD): single peptides in bulk solution

**Figure 1:**
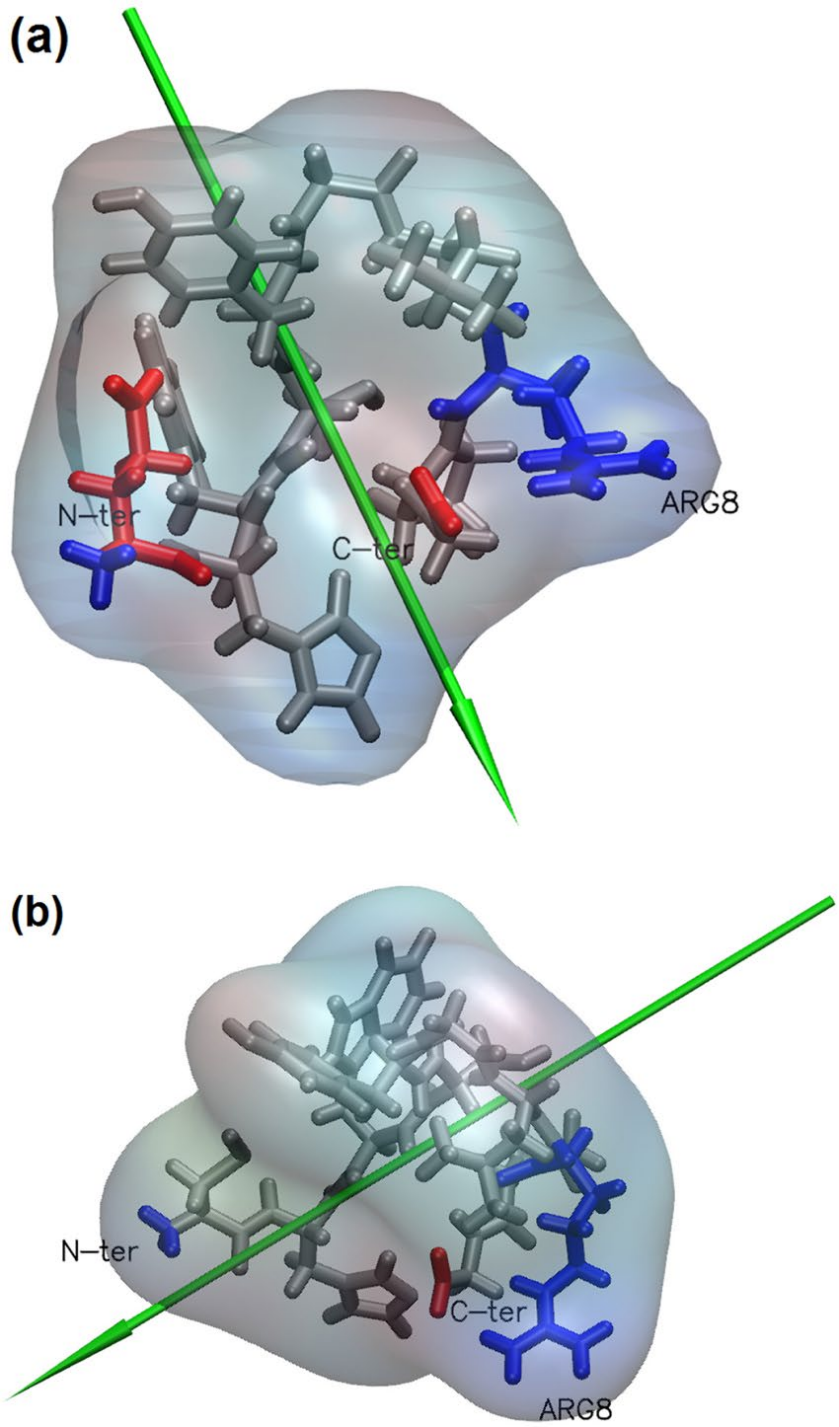
Simulated GnRH-I structures in solution shown using the licorice representation of VMD^38^: (**a**) native GnRH-I with hairpin bend; (**b**) cys-GnRH-I with net positive charge. Positive side chains are blue, negative are red. The green arrows indicate the dipole moment of the peptide.

The GnRH-I and cys-GnRH-I structures in bulk solution, without any surface interaction, are illustrated in Fig. 1. The peptides tend to adopt hairpin bend conformations as shown. Figure 1 also shows the charged residues in the structures. The native GnRH-I comprises a negative glutamic acid (Glu1) residue at the positive N-terminus, a positive arginine (Arg8) and the negative C-terminus, and so has no overall charge at pH 7. In contrast, the cys-GnRH-I has the Glu1 replaced by a neutral cysteine residue. This does not dramatically impact the peptide conformation in bulk solution, but it leads to a change in the overall charge and its distribution along the chain. This will impact how it adsorbs to the silica surface as explained below.

### MD: Single Peptide Adsorption to the Silica Surface

We performed a number of independent simulations for single GnRH-I and cys-GnRH-I adsorbing to the model silica surface. While the simulations differ in details such as the time it takes for the adsorption to occur, they all follow the same pathway to adsorption and have the same peptide-dependent adsorbed conformation that we now describe.

Although the native GnRH-I is neutral, it has a dipole moment running from the charged Arg8 to the negatively charged C-terminus (Fig. 1). Therefore, in the adsorption simulations, GnRH-I oriented in the electric field above the siloxide-terminated surface prior to adsorption. Since the surface field was screened by the counter ions in solution, the adsorption was relatively slow, occurring on a 10 ns timescale in the simulations. This allowed the peptide time to diffuse above the surface and find its preferred orientation before adsorption, a process described elsewhere^22,29^. When the peptide adsorbed, it was steered by Arg8, whose positive side-chain extended towards the negatively charged surface. This steering process enabled the side-chain to penetrate the water layers that formed at the surface, leading to strong adsorption and anchoring at the surface. The crucial role played by Arg in protein adsorption at negatively charged surfaces has been described elsewhere^23,25,30–32^. Subsequently, the rest of the peptide tended to collapse to the surface bound by numerous short-range van der Waals forces, which are weaker than electrostatic forces yet sufficient in number to bind the peptide to the surface. Therefore, the final adsorbed conformation was such that GnRH-I was strongly anchored in its centre by Arg8, but its negative C-terminus was only loosely bound to the surface and so more available for interactions with the solvent environment. The N-terminus is neutral, and so was also loosely bound to the silica surface.

**Figure 2:**
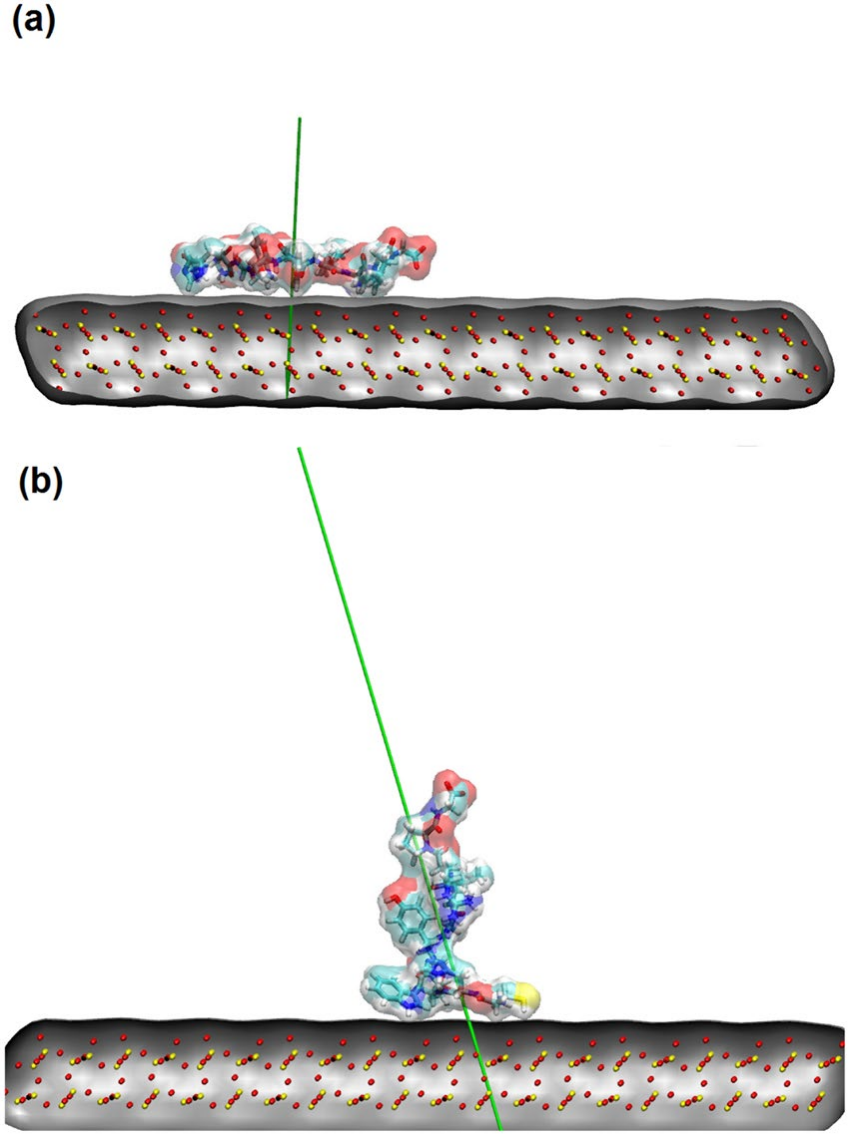
Simulated GnRH-I adsorption to the model silica surface: (**a**) isolated native GnRH-I collapsed to surface, binding through Arg; (**b**) cys-GnRH-I in a metastable upright position, bound at the N-terminus. The peptide is displayed as ‘licorice’ in VMD^38^, with sulphur yellow, oxygen red and hydrogen white, and the green arrow indicate the peptide dipole moments. The silica surface comprises red oxygen and yellow silicon ions. The water and counter ions are not shown for clarity.

A typical adsorption conformation is shown in Fig. 2. We note that due to the fluctuating environment, the conformation could evolve, but the peptide remained bound to the surface with very little centre-of-mass diffusion across the surface apparent in our 50 ns trajectories (data not shown).

In contrast, the cys-GNRH-I peptide has a net positive charge with a dipole moment running from the negative C-terminus to the positive N-terminus (Fig. 1). When this peptide aligned in the electric field above the surface it was elongated normal to the surface. Again this peptide had time to diffuse above the surface before adsorbing due to the screening effect of the counter ions in solution. Indeed, the cys-GnRH-I initially adsorbed in this conformation, with the positively charged N-terminus acting as an initial weak anchor (see Fig. 2). However, this conformation was metastable, with the isolated peptide eventually collapsing to the surface in a manner similar to the native one, only in this case the peptide’s N-terminus was strongly anchored to the silica surface and only the C-terminus was free to interact with the solvent environment.

### MD: Peptide Clusters Adsorbed on the Silica Surface

The different behaviour of the single GnRH-I and single cys-GnRH-I adsorption to the silica surface suggests that the peptides might adopt different conformations in a crowded environment. Experimentally we load the SiNPs with many, rather than just individual, peptides and so we explored this in our simulations. To do this, we created islands of peptides at the surface, and ran the trajectories to explore the stability of the islands and whether the peptide conformation adapts to the presence of other peptides.

**Figure 3:**
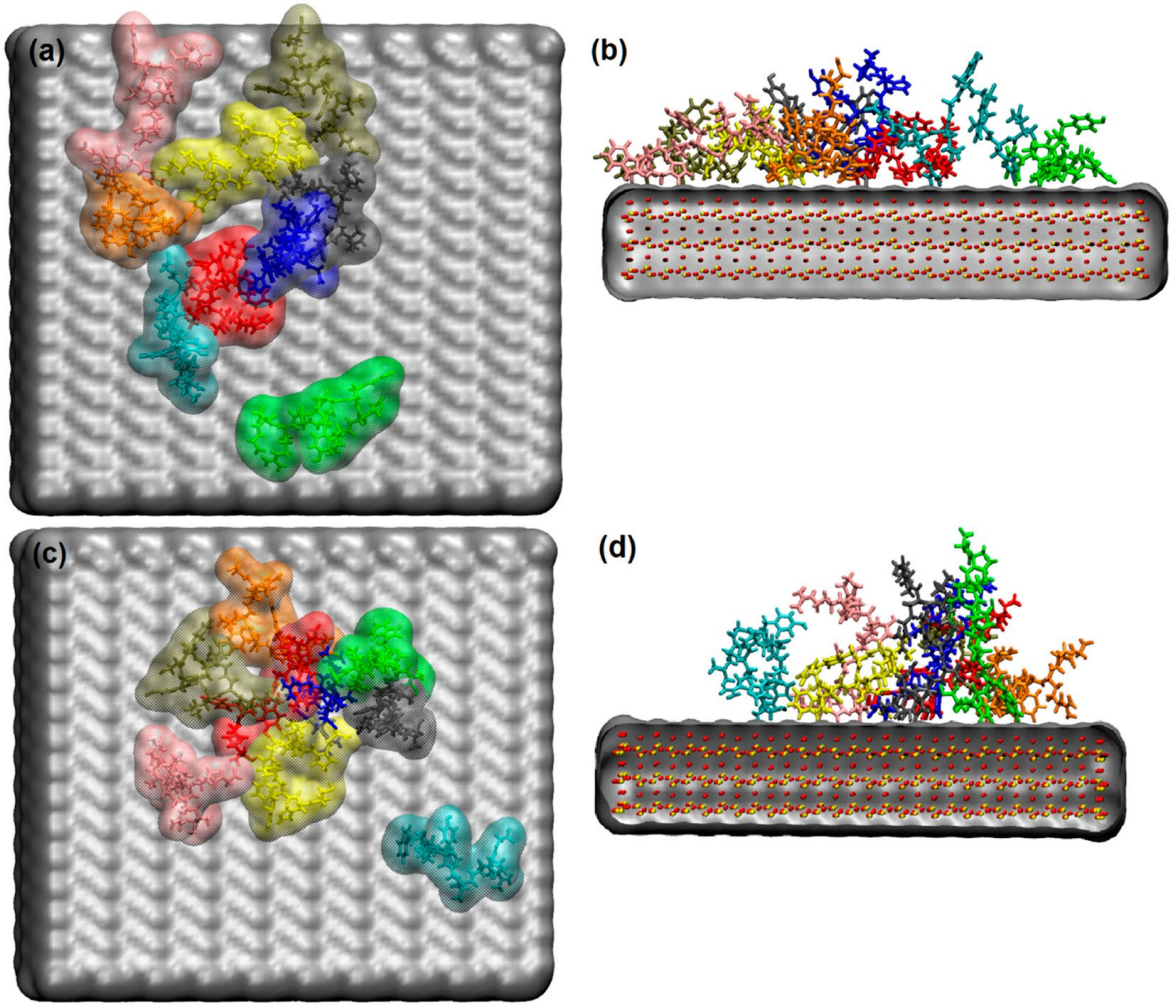
Clustering of GnRH-I at the silica surface: (**a**) native plan view; (**b**) native side-view; (**c**) cys-GnRH-I plan view; (**d**) cys-GnRH-I side view. The peptides are displayed using VMD ‘licorice’, with each peptide given a different colour for ease of identification. The water is not shown for clarity.

In the case of the native GnRH-I, we began with an island of 9 peptides on the surface. Each peptide was given the conformation shown in Fig. 2a, and the peptides were close-packed into a square array on the model silica surface. During the ensuing trajectory, some of the peptides at the edge of the island diffused away to lower the surface density, as can be seen in Fig. 3, which shows the structure after 50 ns. This spreading of the peptide island allowed greater access to the surface for the screening Na^+^ counter ions. Since GnRH-I is neutral, the surface charge density is not compensated by the adsorbed peptide, so the energy of the system is lowered by the separation of the peptides even though this reduces the van der Waals interactions between the peptides. A denser film of neutral peptides would impede the close approach of the counter ions to the surface, and so is energetically less favourable. As a consequence of this electrostatic driving force to keep the peptides separated, the way that the peptides present to the solution remains largely unchanged from the isolated peptide described above, with the N and C termini only loosely bound to the surface and available for interactions with the environment above the surface.

For the cys-GnRH-I island, we began with a close-packed array of 9 peptides with the elongated conformation taken from the isolated peptide adsorption simulations (see Fig. 2b). As shown in Fig. 3, this island tended to be more stable in the subsequent 50 ns trajectory. Here it is apparent that one of the peptides has diffused away from the main cluster, but the remainder have stayed in a coherent island. This configuration is favoured by the electrostatics, since the charged N-terminus of the peptides balances the negative charge of the silica surface, screening it from the counter ions, which are not required here for surface neutralisation. Also apparent in Fig. 3 is that the elongated conformation of the cys-GnRH-I peptides is stabilised within the island, whereas for the isolated peptide it was only metastable. It suggests that cys-GnRH-I will tend to adsorb in dense monolayers at the surface of the SiNPs, preferentially presenting the C-terminus to solution and using the positive N-terminus as the binding site to the surface. This is in contrast to the sparser layer preferred by native GnRH-I, where both termini are presented to the solution by the adsorbed peptide.

### MD: The cys-GnRH-I-BSA Conjugate

**Figure 4:**
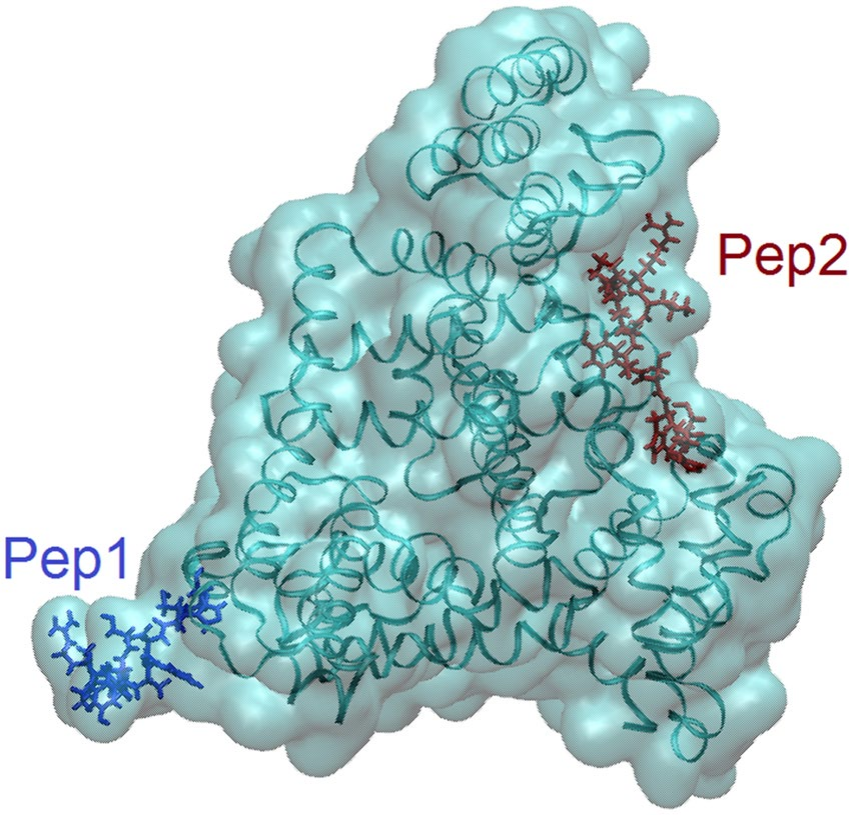
The simulated cys-GnRH-I-BSA conjugate. The dark blue peptide (Pep1) is attached to the BSA via its cysteine residue, and is exposed to solution, whereas the red peptide (Pep2) is attached through its C-terminus glycine and is collapsed onto the BSA surface. The BSA is displayed as a ‘cartoon’ in VMD^38^ and the peptides as ‘licorice’. The water is not shown for clarity.

We simulated a cys-GnRH-I-BSA conjugate in bulk solution, with two cys-GnRH-I peptides conjugated (as per the EDC 1-ethyl-3-[3-dimethylaminopropyl] carbodiimide reaction) to a BSA monomer. The peptides were conjugated to BSA via either the C- or N-terminus respectively. Figure 4 clearly shows that one peptide (red, ‘Pep2’, conjugated using its C-terminus) remains in a very stable position attached to the BSA structure, whereas the other bound peptide (blue, ‘Pep1’, conjugated using the cysteine-tagged N-terminus) extends out from the BSA and interacts more freely with the surrounding environment. This agrees with an earlier observation that conjugation through the N-terminus to a carrier protein is more effective in producing vaccination than through the C-terminus^33^.

The result from the MD simulation is encouraging; it suggests that cys-GnRH-I peptides conjugated to BSA can be free to interact with the surrounding environment. In a 1:1 weight ratio of BSA to peptide, there would be on average 20 peptides free to conjugate to each BSA monomer. Therefore, it appears that there would be various points on the peptide-BSA conjugate that would expose GnRH-I epitopes to solution, presenting them for interaction with the immune system. As we will see below, this correlates well with specific antibody results.

While further simulations should be carried out in order to investigate in detail how a greater number of peptides bound to BSA would present themselves to solution, the results presented here support the interpretation that peptide-BSA conjugation creates useful systems for immunological response. Furthermore, while we do not explicitly simulate the adsorption of the peptide-decorated BSA to the SiNP, previous work^24^ shows that the BSA adsorbs in a specific way while retaining its structure, so that the BSA-nanoparticle systems will retain the exposure of the peptides to solution.

### Immunology Study: Specific Antibody and Testosterone Levels

We performed a series of immunisation studies using the various treatments to the different groups given in Table 1; each dose delivered 50 µg of peptide, and each group comprised 5 animals following previous protocols^8^. We assessed the GnRH-specific antibody and testosterone levels after 13 weeks following fortnightly doses in each treatment group. In this way we aim to understand which treatments are most effective, and to then correlate the efficacy with the insight into the epitope presentation provided by the MD results shown above. We find that all treatments have some immunological impact, demonstrating that the various conjugates remain sufficiently intact *in vivo*.

**Table 1:** Immunisation treatment groups.

Group	Treatment	Delivery System
I	Untreated control	N/A
II	Native GnRH-I	SiNP
III	Cys-GnRH-I	SiNP
IV	BSA-native GnRH-I	None
V	BSA-cys-GnRH-I	None
VI	BSA-native GnRH-I	SiNP
VII	BSA-cys-GnRH-I	SiNP

**Figure 5:**
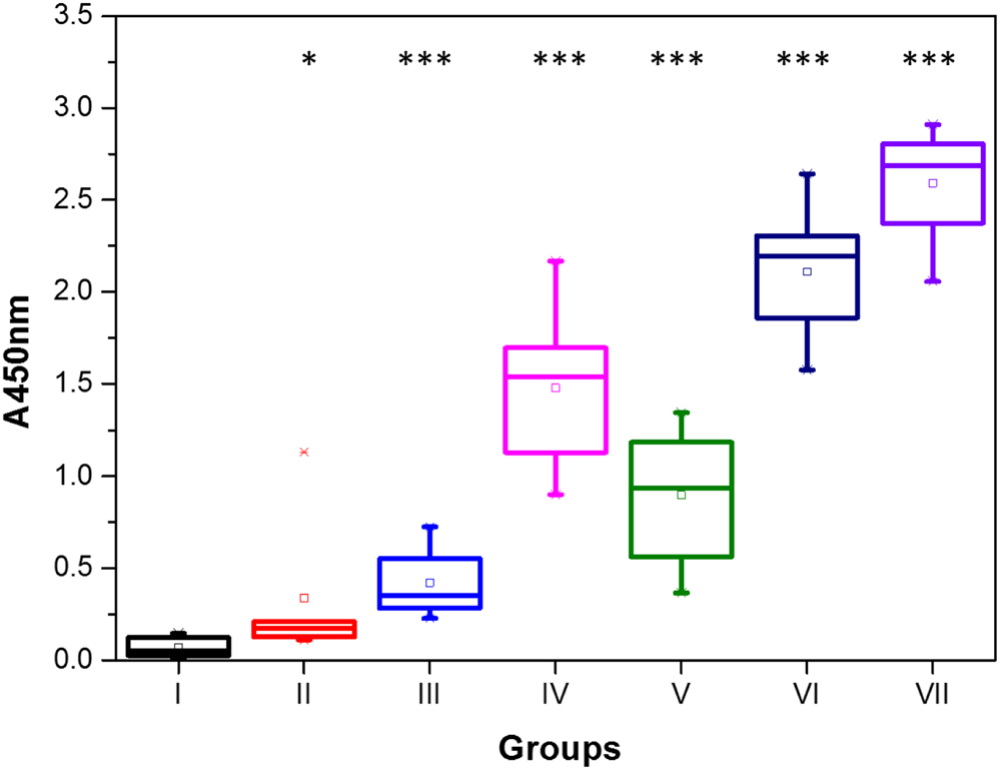
GnRH-specific IgG antibody levels at 13 weeks measured by the absorbance at 450 nm against a standard curve supplied in the testosterone kit. The data are displayed as a box-plot which shows the median, 2^nd^ and 3^rd^ quartile as the horizontal lines of the boxes, the mean as a symbol in the box, and whiskers showing the 9^th^ and 91^st^ percentiles. Outliers are also shown. ***p < 0.001; *p < 0.025 indicates responses significantly higher than the control.

Figure 5 shows the final GnRH-specific IgG response in week 13 for all the groups. Student’s t-test between the treatment groups and the control are used to create the p-values, as routinely done in immunology studies; p-values below 0.001 show a significant increase in antibodies for all the treatments except Group II (Native GnRH-I, SiNP).

The final antibody response for Group VII, the treatment using the BSA-cys-GnRH-I conjugate with SiNP, was much higher (A450 ~2.6 ± 0.3) than any other group, while the Group V antibody level for the BSA-cys-GnRH-I conjugate without the SiNP adjuvant was much reduced (A450 ~0.9 ± 0.4). These antibody titres are similar to those induced in female mice, using a different carrier protein and lipid nanoparticle delivery system^8^.

The treatment groups using the native GnRH-I conjugated to BSA provided a similar response, with the SiNP adjuvants producing higher antibody responses (A450 ~2.1 ± 0.5 for Group VI with SiNP versus 1.5 ± 0.6 for Group IV without).

The antibody responses without the use of the carrier BSA protein were all lower still. Group III (cys peptide with SiNP) produced significant (p < 0.001) results, however the antibody response (A450 ~0.3 ± 0.2) was almost an order of magnitude lower than that of Group VII (cys peptide conjugated to BSA with SiNP). Similarly, Group II (native peptide with SiNP) showed an even lower antibody response, close to baseline, with only marginal statistical significant difference (p < 0.025) in comparison to the control Group I.

These results indicate that the best immunological response is elicited by peptides conjugated to BSA adsorbed onto the SiNPs. The MD simulation results above provide a molecular-scale picture for this, since they show that the conjugates present epitopes to the solution more effectively than when the peptides are directly adsorbed to the SiNPs. The effectiveness of the BSA conjugates is improved when they are adsorbed to the SiNPs, which therefore act as adjuvants for this vaccine.

**Figure 6:**
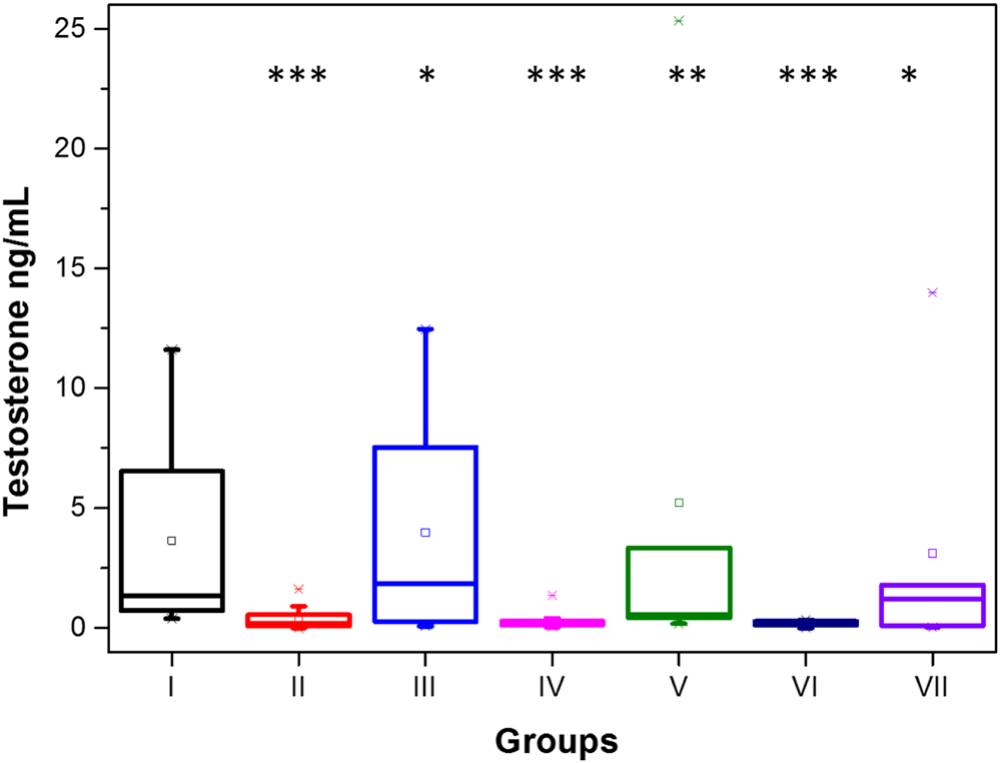
Testosterone levels in serum in study week 13. The data are displayed as a box-plot which shows the median, 2^nd^ and 3^rd^ quartile as the horizontal lines of the boxes, the mean as a symbol in the box, and whiskers showing the 9^th^ and 91^st^ percentiles. Outliers are also shown. *P < 0.9; **P < 0.6; ***P < 0.001.

In Fig. 6, the results for the testosterone levels in study week 13 are shown. All treatments containing the native peptide (Groups II, IV and VI) produced a reduction in testosterone. Surprisingly, and in contrast to the antibody results, the Group II treatment with the native GnRH-I peptide directly adsorbed to the SiNP surface showed the greatest effect in reducing the average testosterone level, with a decrease of ~9.5 ng/ml (>95%, p < 0.001) from that of the control group. In stark contrast, the effects of Group III using cys-GnRH-I adsorbed to SiNPs showed no reduction in the average testosterone levels (p < 0.9). This pattern of behaviour is reproduced with the peptides conjugated to BSA; Groups IV and VI using BSA-native GnRH-I effectively reduced testosterone levels (p < 0.001), while Groups V and VII using BSA-cys-GnRH-I only did so to a much lesser extent (p < 0.6 and p < 0.75 respectively).

In summary, the Group II results appear to be antibody-independent, while the Groups IV, VI and VII are antibody-dependent. The results for Group V indicate that although an antibody response is invoked, it is not high enough to reduce testosterone levels to significant levels compared with the untreated controls.

These results indicate hormonal response is sensitive to the GnRH-I sequence. The MD simulations above show that the native GnRH-I adsorbs to the silica using Arg8, and that the peptide N and C termini are available and exposed to the environment, while still retaining the hairpin loop secondary structure. However, the simulations show that the cys-GnRH-I is bound to the silica surface via its N-terminal residue, which also results in some straightening of its hairpin loop structure. Consequently, the bound native residue is much more able to interact with the GnRH receptor, where terminal residue interactions are key^34^. Additionally, the cys-peptide has one of its terminal residues substituted (glutamic acid for cysteine) so cannot interact with the GnRH receptor as effectively. This also suggests the possibility that the antibodies produced with the cys-GnRH-I have low avidity due to the sequence modification, although previous work^35^ suggests otherwise, and the lack of testosterone knock-down in Group III (Fig. 6) is due to inadequate antibody production (Fig. 5).

Hence, since the native peptide interacts effectively with the GnRH receptor, when it is conjugated to BSA or adsorbed to SiNPs, it may block the receptor. This would prevent free GnRH peptides interacting with the receptor, consequently halting the production of testosterone. This would then account for the results obtained here, with the BSA-cys peptide conjugate systems having reduced impact due to the sequence modification.

#### Immunological Study: Effect on spermatogenesis

At post-mortem, the testes were dissected and examined for the density of spermatozoa, spermatids, germinal epityhelium and quantity of sperm tails in the tubules to quantify the level of spermatogenesis. The scoring system used is defined in Table 2.

**Table 2:** Modified criteria of testicular score count used to quantify level of suppressed spermatogenesis in mice testes^42^.

Scores	Criteria
6	Complete spermatogenesis; characterised by regularly thickened germinal epithelium and a visible lumen; red sperm tail or blue sperm head coverage >50–80% in the lumen.
5	Thickened germinal epithelium with a visible lumen, <50% lumen coverage with dull colored sperm tail
4	Disorganised germinal epithelium, marked sloughing off germ cells and obliteration of the lumen. Presence of degenerating sperm tails in the lumen with 10–15 spermatozoa/spermatids present/tubular sections
3	Few spermatozoa (<5–10) and/or spermatids (<10) present in the tubules. The germinal epithelium is scanty
2	No spermatozoa but few spermatids (<5–10) are present
1	No spermatozoa/spermatids but presence of several or many layers of spermatocytes

Complete azospermia was not seen in any of the testicular sections. However, there was arrest of spermatogenesis in the convoluted seminiferous tubules at score level 5 as shown in Table 3. The highest rate of suppression was seen in case of SiNP-BSA-cys-GnRH-I treated animals (Group VII). Testicular spermatogenesis was mostly suppressed at score level 5 and the lowest in Groups I (untreated control) and III (SiNP-cysGnRH-I).

**Table 3:** Testicular score counts using the criteria of Table 2.

Treatment groups	Number of views at a particular score					
	6	5	4	3	2	1
I Control	21	19	9	1	—	—
II SiNP-GnRH-I	18	22	7	2	1	—
III SiNP-Cys-GnRH-I	19	22	8	1	—	—
IV BSA-GnRH-I	16	27	5	1	1	—
V BSA-Cys-GnRH-I	16	23	7	3	1	—
VI SiNP-BSA-GnRH-I	18	25	5	2	—	—
VII SiNP-BSA-Cys-GnRH-I	15	22	9	2	1	1

The clearest indication of efficacy was obtained by examining the percentage number of views attaining a score of 3 or less in a particular treatment group. Groups I and III only showed 2% of views with these scores. In comparison Groups IV and VI showed 4%, Group II 6%, and Groups V and VII 8%, with only Group VII showing a score of 1.

These results indicate that the Group VII treatment was the most effective at arresting spermatogenesis. This is understandable in light of the effective antibody production alongside the testosterone reduction in this group. However, all treatments had some impact on the spermatogenesis, the only exception being Group III; Figs 5 and 6 show that Group III is ineffective in both producing antibody and reducing testosterone.

## Discussion and Conclusions

In this study we have explored the use of spherical, non-porous 200 nm Stöber SiNPs as adjuvants for self-peptide vaccines. The experimental work has been supported and enhanced by biomolecular simulations that provide molecular-scale understanding of the vaccine systems, allowing the results to be interpreted with a physical insight that is otherwise unattainable.

It was found that immunisation against the native GnRH-I peptide adsorbed to SiNPs generated a poor immune response. This is perhaps due to the collapsed nature of the native peptide on the silica surface revealed by the simulations, thus resulting in the peptide being unavailable to interact with antigen presenting cells *in vivo* and therefore limiting the specific immune response. In contrast, the cys-GnRH-I adsorbed onto the SiNPs elicits a specific antibody response. Our simulations show that this peptide interacts with the silica surface via the N-terminal residue (cysteine) and ‘stands’ in a crowded peptide layer; therefore it is free to interact with the environment. If the peptide can interact with antigen presenting cells, it explains the generation of a specific antibody response.

Conjugation of either the native GnRH-I or cys-GnRH-I to BSA results in an improved antibody response. In these formulations, the peptides are more likely to be exposed to the surrounding environment *in vivo*, as is apparent from our simulations. It should also be noted that the addition of SiNPs to these formulations enhances the specific antibody response, so that the SiNP is an effective adjuvant for this vaccine.

The antibody effects of immunisation against these peptides appear to be in stark contrast to the hormonal effects. Mice treated with formulations including the native peptide all exhibited a marked decrease in testosterone production. Again we understand this effect in light of the simulation results. Since the terminal residues play a key role in the peptide interactions with the GnRH receptor, it is clearly favourable to retain the native sequence at the termini. Furthermore, systems that leave the termini free to interact with the external environment will be the most effective in blocking the receptors, and the simulations show that the native GnRH-I SiNP formulation fulfils this requirement.

Arrest of spermatogenesis in the treatment groups correlates with the production of antibody or reduction in testosterone. Group VII, the treatment with cys-GnRH-I conjugated to BSA and adsorbed to the SiNP, was the most effective, whilst Group III with cys-GnRH-I directly adsorbed to SiNP was ineffective.

It is therefore apparent that we can, with some success, interpret and understand the immunological responses to the vaccine systems by employing suitable molecular simulations. In order to successfully design and formulate peptide-based vaccines, the interactions between antigen and adjuvant have to be fully understood in order to produce an effective and stable product. The use of MD simulations allows these interactions to be studied in detail; we therefore believe that this combined modelling and experimental approach can facilitate the development of new vaccines while greatly reducing the experimental load and costs, as well as providing a predictive tool that can lower the numbers of animals used in pre-clinical investigations of peptide vaccines.

## Materials and Methods

### Molecular Dynamics Simulations

Our computational simulations follow the methodology outlined in our previous work^29^. We used NAMD2.8^36^ with the CHARMM27 force-field and the TIP3P water model. We employed periodic boundary conditions, the Particle Mesh Ewald for the electrostatic interactions^37^ and a cut-off of 12 Å for short-range forces. Analyses of the trajectories were performed using VMD^38^.

The model silica surface was created from a $\{11\bar{1}\}$ oriented slab of alpha-crystabolite terminated to create a dipole moment from the positive Si-rich surface to the O-rich one; the latter presents siloxide groups to the water above it, modelling the surface chemistry observed experimentally^5^. Furthermore, the use of periodic boundary conditions with such a slab creates an electric field above the surface, again in line with experimental observations for Stöber SiNPs. This electric field is screened by the addition of NaCl ions to the water; here we use a silica slab of dimensions 86 Å × 80 Å × 13 Å with 88 Na^+^ ions to screen the electric field above the siloxide-rich surface, and 88 Cl^−^ ions to screen the positively charged Si-rich surface on the opposite side of the slab.

The native GnRH-I structure used was 1YY1.pdb^39^ and the cys-GnRH-I peptide was a modified version of 1YY1.pdb where the N-terminal glutamic acid residue was changed to a cysteine residue. The residue protonation was taken to be that at pH 7. The adsorption simulations were prepared with one peptide 20 Å above the target siloxide-rich surface, plus a distribution of the Na^+^ and Cl^−^ ions, and the system was solvated in a water box that extended at least 15 Å away from any protein atom. The resulting systems contained ~80,000 atoms. Each simulation was preceded with an equilibration and minimisation period, with a production trajectory of 50 ns using a time-step of 1.0 fs. To reduce the computational time all bonds and angles in the water molecules were constrained.

The BSA-peptide system was prepared by adding the cys-GnRH-I at suitable surface sites on the BSA. We mimicked the covalent bonding of the peptide N-terminus to Phe308, and the C-terminus to Leu115. The MD trajectory for the solvated BSA-peptide system, containing ~180,000 atoms, was simulated for a duration of 50 ns at a constant temperature of 27 °C.

### Materials Preparation

SiNPs were prepared using the Stöber synthesis method^40^. Tetraethylorthosilicate (TEOS, 99%, Aldrich Chemical Co.), ethanol (EtOH, 99.9%, Fisher Scientific) and ammonium hydroxide (28.0–30.0%, Sigma-Aldrich) were used as initial reactants for this synthesis. A peristaltic pump was used to control the addition rate of the TEOS/EtOH mixture.

**Figure 7:**
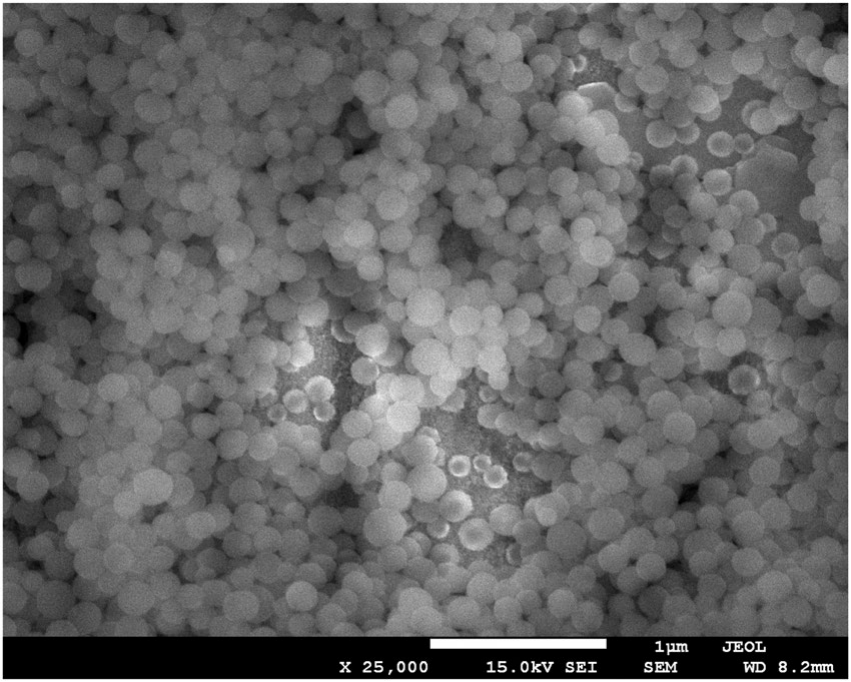
Scanning electron microscope image of the silica nanoparticles manufactured in this study. The scale bar is 1000 nm.

The resulting nanoparticulate solution was centrifuged 3 times at 8,000 RPM and re-dispersed. It was stored in a 1:1 water/ethanol solution with a concentration of 0.2 g/mL silica. Figure 7 shows a scanning electron microscope (SEM) image of the SiNPs manufactured in this study; a small sample of the suspension was dried on a support for this image. It shows that the nanoparticles are approximately spherical with a diameter ~200 nm with close homogeneity. Dynamic Light Scattering (DLS) measurements (not shown) confirm this, finding a mean diameter of 193 nm at pH 8.7 (polydispersity index, PDI, of 0.079) to 199 nm at pH 4.0 (PDI of 0.090).

Native GnRH-I (EHWSYGLRPG, 90% purity, synthesised by Genosphere Biotech, France) and a N-terminal modified GnRH-I peptide, cys-GnRH-I (CHWSYGLRPG, 90% purity, synthesised by Immune Systems Ltd, UK), were used in this study. BSA and ovalbumin were purchased from Sigma-Aldrich Company Ltd, UK.

The immunogens consisted of either free peptides or peptide bound to BSA. Where the peptide was conjugated to BSA, each peptide was conjugated in a 1:1 weight ratio using the 1-ethyl-3-[3-dimethylaminopropyl] carbodiimide (EDC) reaction (Sigma-Aldrich Company Ltd, UK) and separated using 30 K Merck Millipore filtration tubes. The GnRH-I peptides (native and cys-GnRH-I) bound to ovalbumin were used as enzyme-linked immunosorbent assay (ELISA) plate coating antigens and were prepared via the same EDC reaction following Oonk *et al*.^41^.

### Immunisation

All animal experiments were conducted in accordance to UK Home Office Legislation and have been approved by the University of Strathclyde Ethical Review Committee. We used 5 animals in each treatment group and no repeats, in line with the Home Office project licence guidelines to minimise the use of animals. In-house bred male BALB/c mice, aged 8 weeks at the start of the study, were kept in a controlled environment: room temperature 22 °C; 50–70% humidity; summertime light cycle of 14 hours light per day; 5 animals per cage; one cage per treatment group. All mice were ear coded; food and water were supplied *ad libitum*. Subcutaneous injections were administered fortnightly; the mice were sacrificed after 13 weeks and bloods taken. The immunisation treatment groups are provided in Table 1.

The Group II treatment was formulated by mixing 100 µL SiNPs (0.2 g/ml) and 900 µL deionised water. Native GnRH-I (1.0 mg) was dissolved in 1 mL deionised water and added to this solution. The resulting mixture was stirred at room temperature for 1 h and then frozen at −20 °C until further use. The Group III treatment followed the same procedure, using cys-GnRH-I.

The treatment for Group IV was prepared by dissolving native GnRH-I and BSA in deionised water in equal weights (the final sample containing 2.5 mg peptide). The BSA and peptides were then conjugated using the EDC reaction: the native GnRH-I was mixed with the BSA solution and stirred using a magnetic stirrer for 30 min at room temperature. EDC (50 mg) was dissolved in 1 mL deionised water. This was then added slowly to the protein/peptide mixture and stirred for 6 h. In order to remove excess EDC, the mixture was filtered using 30 K Merck Millipore filters at 3500 RPM for 15 min. The resulting solution was then frozen at −20 °C until further use. Group V’s treatment was prepared in the same way using cys-GnRH-I. Group VI and VII used the conjugated native and cys-GnRH-I solutions respectively, mixing them with 100 µL SiNPs (0.2 g/ml) +900 µL deionised water and then frozen at −20 °C to await further use.

In all immunisation treatment groups, each mouse was fortnightly administered a subcutaneous dose of 0.1 mL formulation, containing the equivalent of 50 µg of peptide per dose. After 13 weeks, 7 days after the final administration, blood was collected via cardiac puncture. Each blood sample was centrifuged at 1000 g for 20 min. The serum was then collected in fresh microfuge tubes and frozen at −20 °C until analysed by ELISA.

### Measurement of GnRH-specific Antibody and Testosterone Levels

All wash steps were carried out three times with phosphate buffered saline (PBS), pH 7.4, containing 0.01% (v/v) Tween 20 (PBS-Tween) and incubations carried out for 1 h at 37 °C unless otherwise stated. A tissue culture grade 96-well plate was coated with the corresponding GnRH-I peptide-ovalbumin conjugate and the ELISA carried out as described by Gebril *et al*. (2014)^8^. The absorbance at 450 nm of each plate was read on a SpectraMax M5 plate reader (Molecular Devices, USA).

Serum testosterone levels were measured using a testosterone ELISA kit (Alpha Diagnostic International USA) according to the manufacturer’s instructions. All mouse sera samples were assayed in duplicate and low and high testosterone control samples were included in all runs.

### Evaluation of effect on Spermatogenesis

At post-mortem, the testes were dissected and preserved in formalin, and tissues were sectioned^42^. A total of fifty tubular cross sections per testes was examined by light microscopy for the densities of spermatozoa, spermatids, germinal epithelium and quality of sperm tails in the tubules to quantify the level of spermatogenesis. The criteria in Table 2 was used to provide a scoring system.

## Data Availability

Supplementary Materials: run files for the MD simulations reported here are available at 10.15129/70dc12db-5f73-4603-8242-80202c99f7be.
